# Suppression of Oxidative Stress as Potential Therapeutic Approach for Normal Tension Glaucoma

**DOI:** 10.3390/antiox9090874

**Published:** 2020-09-16

**Authors:** Chikako Harada, Takahiko Noro, Atsuko Kimura, Xiaoli Guo, Kazuhiko Namekata, Tadashi Nakano, Takayuki Harada

**Affiliations:** 1 Visual Research Project, Tokyo Metropolitan Institute of Medical Science, 2-1-6 Kamikitazawa, Setagaya-ku, Tokyo 156-8506, Japan; harada-ck@igakuken.or.jp (C.H.); noro-tk@igakuken.or.jp (T.N.); guo-xl@igakuken.or.jp (X.G.); namekata-kz@igakuken.or.jp (K.N.); harada-tk@igakuken.or.jp (T.H.); 2 Department of Ophthalmology, The Jikei University School of Medicine, 3-25-8 Nishi-Shimbashi, Minato-ku, Tokyo 105-8461, Japan; tnakano@jikei.ac.jp

**Keywords:** glaucoma, retinal ganglion cell, optic nerve, oxidative stress, neuroprotection, glutamate transporters, ASK1, marmoset, drug repositioning, food factor

## Abstract

Glaucoma is a neurodegenerative disease of the eye, which involves degeneration of retinal ganglion cells (RGCs): the output neurons of the retina to the brain, which with their axons comprise the optic nerve. Recent studies have shown the possible involvement of oxidative stress in the pathogenesis of glaucoma, especially in the subtype of normal tension glaucoma. Basic experiments utilizing rodent and primate models of glaucoma revealed that antioxidants protect RGCs under various pathological conditions including glutamate neurotoxicity and optic nerve injury. These results suggested that existing drugs and food factors may be useful for prevention and hence therapy of glaucoma. In this review, we highlight some therapeutic candidates, particularly those with antioxidant properties, and discuss the therapeutic potential of RGC protection by modulating gene expressions that prevent and ameliorate glaucoma.

## 1. Introduction

Glaucoma is a neurodegenerative disease of the eye and it is one of the major causes of blindness. It is usually associated with sustained elevation of intraocular pressure (IOP), damage to the optic nerve, and death of retinal ganglion cells (RGCs). The cell body of RGCs is located in the retina and they project their axons to the brain nuclei through the optic nerve. To date, more than 40 subtypes of RGCs have been identified and how they respond to injury has been studied in various models [1,2,3,4]. Glaucoma therapy mainly focuses on reducing IOP and this approach prevents or slows down disease progression. However, the therapeutic effect of this method alone is not sufficient for some patients and disease progression continues despite treatment. In addition, there is a form of glaucoma that shows glaucomatous optic neuropathy without elevation of IOP, termed normal tension glaucoma (NTG). These suggest that there are factors other than high IOP that could be a therapeutic target for glaucoma. One of the factors that was focused upon previously was excitotoxicity, but it is known now that the glutamate level in the vitreous of glaucoma patients is not upregulated; although, glutamate neurotoxicity may still play a part in the pathology of glaucoma [5,6]. Pathogenesis of glaucoma is complex, and it involves multiple factors. Oxidative stress is one of the risk factors for glaucoma and the level of glutathione (GSH), a major antioxidant in the retina [7], is decreased in the glaucoma patient plasma [8,9]. Moreover, a recent report demonstrated that oxidative stress is increased in the common marmoset with pathological features of glaucoma [10]. Studies using animal models indicate that suppression of oxidative stress increases RGC survival [11,12,13,14,15], suggesting that antioxidants are potential candidates for glaucoma therapy. In this review, we discuss recent works on the role of glutamate transporters in glaucoma patients, the effects of antioxidants in mouse models of glaucoma, and characterization of glaucomatous marmosets.

## 2. Identification of Sequence Variants in EAAT1/GLAST in Glaucoma Patients

We previously reported that deletion of glutamate/aspartate transporter (GLAST) shows NTG-like pathological features, including RGC death and optic nerve degeneration after birth with a normal level of IOP [16]. GLAST is expressed in Müller glia in the retina and removes excess glutamate from the synapses, thus preventing excitotoxic damage on surrounding retinal neurons [17], and the level of GLAST is decreased with aging, especially in glaucoma patients [18,19]. In addition, the antioxidant GSH is synthesized from glutamate, cysteine and glycine, and in GLAST knockout (KO) mice, decreased glutamate uptake into the Müller glia leads to reduced GSH level [16], indicating that GLAST KO mice demonstrate some pathological features of NTG and that GLAST may be involved in pathogenesis of NTG.

To explore the possibility that loss-of-function mutations in the *GLAST* gene is associated with susceptibility of glaucoma, we performed targeted sequencing of the *EAAT1* gene (the human homolog of *GLAST*) in glaucoma patients [20]. Interestingly, the systematic mutation screening detected four heterozygous mutations that caused amino acid substitutions in the EAAT1 protein in glaucoma patients. The identified mutations are A169G, E219D, T318A, and A329T. We found that 1.6% of glaucoma patients (7 of 440 patients) were heterozygous for these missense mutations, compared with only 0.22% in control subjects (1 of 450 controls). Unfortunately, the parents of all seven patients were deceased; therefore, it remains unknown if they were also affected. In addition, four synonymous variants were identified in 13 patients: c.408G > A, p.V136V in one patient; c.945C > T, p.A315A in 10 patients and one control; c.954C > T, p.T318T in one patient; and c.1448C > T, p.H496H in one patient.

**Figure 1:**
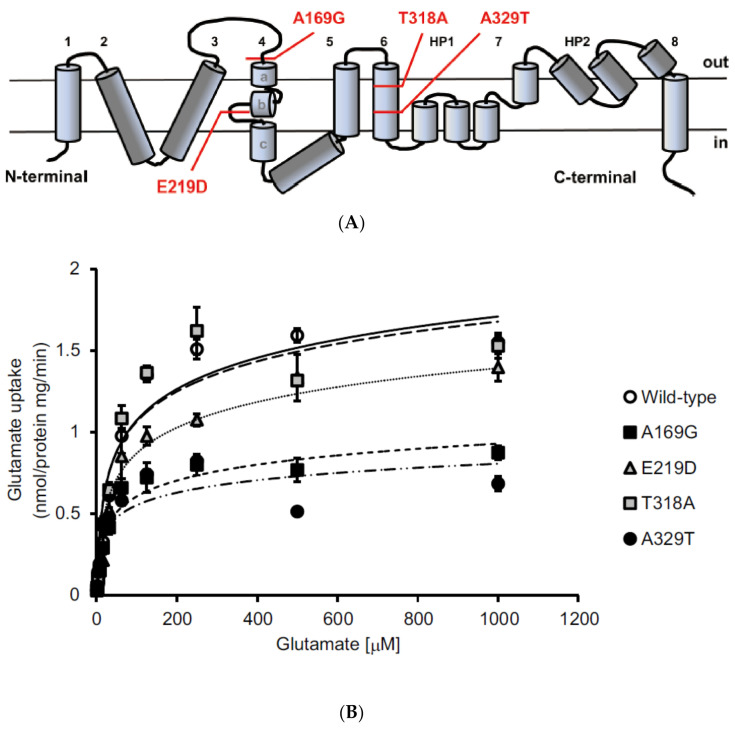
*EAAT1* variants identified in patients with glaucoma and the functional impact of missense mutations on EAAT1 protein functions. (**A**) The predicted topology of the EAAT1 protein showing transmembrane domains 1–8 and re-entrant hairpin loops (HPs) 1 and 2, which flank transmembrane domain 7. The locations of all missense mutations found in the study by Yanagisawa et al. are illustrated. (**B**) Saturation curves for ^3^H-glutamate uptake into human embryonic kidney (HEK) cells transfected with wild-type *EAAT1* or the missense variants (A169G, E219D, T318A, and A329T). Each data point corresponds to the mean and s.e.m. of three individually transfected wells. Representative results of three separate experiments are shown. Reproduced from Yanagisawa et al. [20].

The A169G mutation is located in the extracellular loop that stretches between the third and fourth transmembrane domain and the E219D mutations is located in the loop between fourth transmembrane segments 4b and 4c of EAAT1 (Figure 1A). The other two mutations T318A and A329T are located in the sixth transmembrane domain of EAAT1. The affected amino acid positions are evolutionarily conserved among mammals, suggesting that these amino acid residues are important for EAAT1 function, and that the identified missense mutations may be pathogenic. To test the functional consequences of these missense variants, we measured glutamate uptake activity in human embryonic kidney (HEK) 293T cells transfected with either the wild-type cDNA construct or the mutant expression constructs. We found that the maximum velocity of glutamate transport was significantly reduced in cells expressing A169G or A329T, while no effects were observed with cells expressing E219D and T318A, compared with wild-type EAAT1 (Figure 1B). These results suggested that the A169G and A329T mutations impaired glutamate uptake, whereas the E219D and T318A mutations did not have much effect on EAAT1 function. 

The decreased transporter activities caused by A169G or A329T mutations may be due to changes in impaired cell surface expression of the transporter. To this end we examined the amount of cell surface expression of EAAT1 in the presence or absence of A169G or A329T mutations, and found that introducing the A329T mutation caused a 32.5% reduction in the surface expression of EAAT1, while the A169G mutation did not have any effects. These results suggested that the A329T mutation impairs cell surface expression of EAAT1, resulting in reduced glutamate transport activity. On the other hand, A169G mutation causes functional impairment by means other than affecting cell surface expression of EAAT1, for example, by changing the biophysical properties during the uptake process.

We established that A169G and A329T mutations impair glutamate uptake activity of EAAT1, but does this cause RGCs to die? To confirm this, we examined glutamate-induced RGC death in a mixed culture with GLAST-deficient Müller glial cells transfected with plasmids containing wild-type, A169G or A329T mutations. Reduced glutamate uptake by Müller glial cells was observed with A169G or A329T mutations and RGC death was significantly increased when RGCs were co-cultured with GLAST-deficient Müller glial cells transfected with either A169G or A329T mutants. These data demonstrated that missense mutations of A169G and A329T in *EAAT1* increased the rate of RGC death induced by glutamate neurotoxicity.

Intriguingly, a study by another group reported a lack of association between *EAAT1* gene polymorphisms (single nucleotide polymorphisms; SNPs) and NTG [21]. It is generally assumed that risk alleles with large effect sizes are rare in frequency and are hard to detect using common SNPs [22]. Our study attempted to identify rare variants with large effect sizes by resequencing the coding exons and the intronic boundaries of *EAAT1* in patients with glaucoma and control subjects. Our results suggest that the missense variants are associated with primary open-angle glaucoma (POAG) and with IOP that is within the accepted normal range. All patients with the A169G or A329T variants did not possess *MYOC*, *OPTN*, and *WDR36* mutations. These data collectively suggested that EAAT1 at A169G and A329T mutations may be susceptible genes for POAG, and especially for NTG, and that EAAT1/GLAST is a potential therapeutic target for the treatment of glaucoma.

## 3. Animal Models of Normal Tension Glaucoma

### 3.1. Glutamate Transporter Deficient Mice

GLAST KO mice have been very useful for evaluating the effectiveness of a therapeutic strategy for glaucoma [12,23,24,25,26]. Recent studies examined the effects of astaxanthin, a natural marine carotenoid that is a strong antioxidant, on GLAST KO mouse retinas by evaluation of retinal morphology using spectral domain optical coherence tomography (SD-OCT) [27]. Dietary intake of astaxanthin suppressed the thinning of the ganglion cell complex (GCC) in GLAST KO mice and electron microscopic analysis demonstrated that astaxanthin treatment reduced thinning of the retinal nerve fiber layer in GLAST KO mice [27], suggesting the benefits of use of antioxidants for glaucoma therapy.

Excitatory amino-acid carrier 1 (EAAC1; EAAT3) is another type of glutamate transporter and it is mainly localized to RGCs in the retina. In EAAC1 KO mouse retinas, high levels of oxidative stress markers are observed compared with wild-type mice; and in cultured RGCs from EAAC1 KO mice, susceptibility to H_2_O_2_ insults is higher compared with wild-type RGCs [16,28,29]. Like GLAST KO mice, EAAC1 KO mice reproduce some aspects of sporadic, age-dependent NTG pathology and make a good mouse model of NTG [16]. However, there are some limitations to using these mice as glaucoma models. For example, in human glaucoma, RGC loss is region-specific, but it is distributed across the entire retina in GLAST/EAAC1 KO mice and in some mouse models of ocular hypertension-induced glaucoma [30,31]. Moreover, in human glaucoma progressive retinal degeneration occurs slowly over years, but in GLAST/EAAC1 KO mice, it starts at 3~5 weeks of age, and in GLAST heterozygous mice, it occurs more slowly (1~4 months) and the effect of a drug can be studied over a year [16,32]. Regardless of such limitations, GLAST/EAAC1 KO mice all develop NTG-like phenotypes in a consistent time-course without affecting non-RGCs in the retina [16,29] and have been useful for providing potential therapeutic targets. We demonstrated the therapeutic effects of decreasing oxidative stress in these mice and we recently reported that some widely prescribed drugs suppressed RGC death in these mice without altering IOP. We will summarize these findings in a later section. 

### 3.2. Aged Marmosets Present with Naturally Occurring NTG

In humans, the lamina cribrosa (LC) is considered to be a putative site of optic nerve damage that causes characteristic pathology of glaucoma, but this tissue is absent in mice. The common marmoset (*Callithrix jacchus*) is a small new world primate and use of this animal is increasing particularly in neuroscience research. The brain and eyes of the common marmoset are structurally well developed, including the presence of the LC. Use of this animal has many advantages over use of other non-human primates because the common marmoset has a high reproduction rate for a primate (gestation period of about 5 months and multiple births are common), they reach sexual maturation early (12 to 18 months of age), and they are easier to handle and breed in laboratories. Common occurrence of multiple births is particularly useful as it allows direct comparison of the effects of treatment and placebo between littermates. Furthermore, their compact lifespan enables aging research to be conducted in a relatively short period of time.

#### 3.2.1. Glaucomatous Characteristics in Marmosets are Similar to Human Glaucoma 

**Figure 2:**
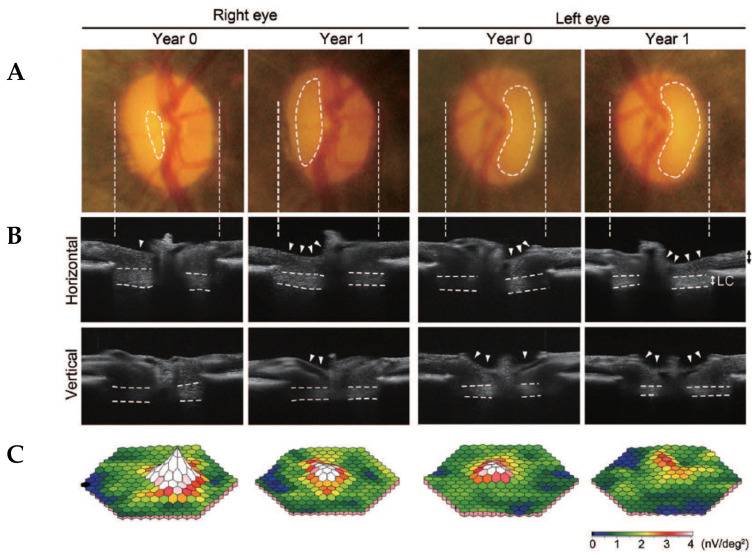
Follow-up studies of a glaucomatous marmoset (13 years old) over 12 months. (**A**) Ocular fundus photographs of initial examination (Year 0) and one year later (Year 1) in the glaucomatous marmoset. Dotted lines indicate the cupping of the optic disc. (**B**) In vivo imaging of the optic disc by the horizontal and vertical scan through the center of the optic disc by spectral-domain optical coherence tomography (SD-OCT). Arrowheads indicate the cupping of the optic disc and dotted lines indicate the lamina cribrosa (LC). (**C**) Three-dimensional plots of the retinal responses as examined by multifocal electroretinogram. Values are given in nV per square degree (nV/deg^2^). Reproduced from Noro et al. [10].

We have recently reported that 11% of aged marmosets show glaucoma-like retinal and brain degeneration as well as the thinning of the LC [10]. The identified glaucomatous marmosets had no genetic mutations in glaucoma-associated genes with a normal IOP level, suggesting that they present with naturally occurring NTG. To minimize animal sacrifice, we used several *in vivo* imaging techniques, including SD-OCT, multifocal electroretinogram and magnetic resonance imaging (MRI), for assessment of glaucomatous pathology in marmosets and followed up disease progression (Figure 2).

The follow-up study of a glaucomatous marmoset after 12 months revealed that its non-diseased (right) eye had also developed glaucoma-like features. Fundus imaging of the left eye at Year 0 detected optic disc cupping and vascular abnormalities (bending and relocation) around the cupping region, but the right eye appeared normal (Figure 2A). One year later at Year 1, the pathological features in the left eye were more exaggerated and we found that the right eye also developed glaucoma-like features. In vivo imaging with SD-OCT captured clear exacerbation in the optic disc cupping over 12 months in the left eye and development of glaucoma-like characteristics in the right eye (Figure 2B). Furthermore, reduction in the thickness of the GCC and LC progressed in both eyes but at different rates. The multifocal electroretinogram also demonstrated progressive decline in visual function over one year, in a same manner as the findings from SD-OCT (Figure 2C). These data indicated that this marmoset presents with binocular glaucoma-like degeneration. Such laterality is also observed in human glaucoma and this discovery is important particularly from the viewpoint of experimental research, because the eye that is going to develop glaucoma can be used to evaluate if a novel therapeutic approach is capable of preventing disease onset or progression.

In vivo imaging with MRI and voxel-based morphometry (VBM) from this study demonstrated significant volume loss in the primary visual cortex (V1) of the glaucomatous marmosets compared with control marmosets. Strikingly, these profiles are almost identical to those observed in advanced POAG patients [33]. Histological analyses showed that the cell number in the fourth layer of the primary visual cortex was significantly less in glaucomatous marmosets than in control marmosets. These data demonstrated atrophy of the central visual system in glaucomatous marmosets by in vivo imaging and histological examinations.

#### 3.2.2. Oxidative Stress is Increased in Glaucomatous Marmosets

**Figure 3:**
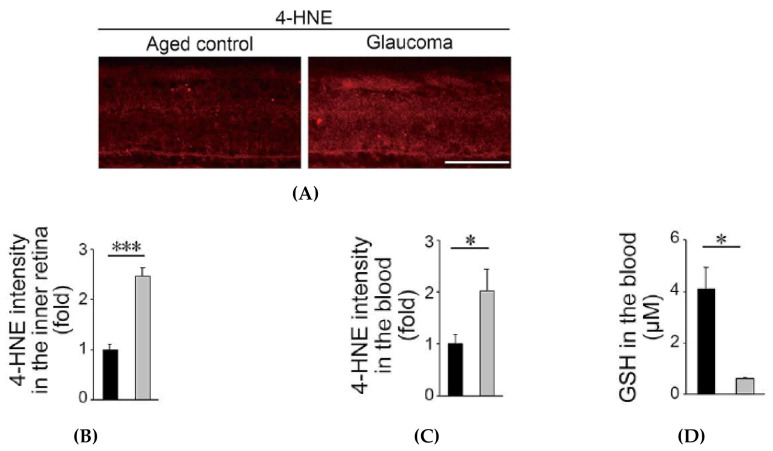
Increased oxidative stress in glaucomatous marmosets. (**A**) 4-hydroxynonenal (4-HNE) expression in the retina detected by immunohistochemistry. Scale bar: 100 μm. (**B**) Quantitative analyses of the intensity of 4-HNE. *n* = 3 per group. (**C**) 4-HNE expression in the blood detected by immunoblot analyses. *n* = 9 (aged) and 3 (glaucoma). (**D**) GSH concentrations in the blood. *n* = 4 (aged) and 3 (glaucoma). The data are presented as means ± s.e.m. *p < 0.05; ***p < 0.001. Reproduced from Noro et al. [10].

Further analyses from this study showed that retinal expression of an oxidative stress marker, 4-hydroxynonenal (4-HNE), was very high in glaucomatous marmosets, particularly in the inner retina (Figure 3A,B). Similarly, the blood 4-HNE expression was significantly higher in glaucomatous marmosets than in control marmosets (Figure 3C). In addition, the level of the antioxidant GSH in blood is reduced in in glaucomatous marmosets. (Figure 3D). Systemic oxidative stress levels are associated with reduced ocular blood flow in NTG patients [34,35,36], and ocular blood flow was also decreased in glaucomatous marmosets. Our data suggest the presence of increased oxidative stress and decreased blood flow in glaucomatous marmosets, similar to human glaucoma.

Unfortunately, the incidence of these naturally occurring NTG marmosets may be too low and the time-course for disease development may be too long to make an effective animal model for testing therapeutic interventions. With much aging research using non-human primates, if it takes decades before diseases are detectable, one study could extend beyond a typical scientific career. In addition, such long-term studies will incur high costs, for example, maintenance for non-human primates requires specialized facilities and staff. Therefore, generating genetically modified marmosets with early onset of disease as a marmoset model of glaucoma may be ideal. Excitingly, generation of transgenic marmosets was first reported in 2009 [37,38]. Based on our studies demonstrating that the loss of glutamate transporters in mice leads to phenotypes similar to NTG [16], we are targeting these genes to generate marmoset models of NTG. Genetic manipulation of the common marmoset raises high hopes for greater understanding of disease pathogenesis and major advances in medical research, which will no doubt provide a beneficial outcome for public health.

## 4. Effects of Suppression of Oxidative Stress in Rodent Models of NTG

Research into the therapeutic effects of reducing oxidative stress on retinal diseases including glaucoma is growing [39]. Here, we describe findings from some of the studies focusing on those using rodent models of NTG.

### 4.1. Apoptosis Signal-Regulating Kinase 1

Apoptosis signal-regulating kinase 1 (ASK1) is a member of mitogen-activated protein kinase that plays important roles in cellular responses to oxidative stress and endoplasmic reticulum stress [40,41]. ASK1 plays an essential part in oxidative stress-induced apoptosis through activation of the ASK1-JNK/p38 pathway [42,43]. Therefore, blocking the ASK1 pathway may be useful to prevent neuronal cell death in various neurodegenerative diseases. We have previously reported neuroprotective effects of *ASK1* gene deletion on RGCs in several different mouse models of glaucoma, including retinal ischemia, optic nerve injury (ONI) and GLAST KO mice (GLAST/ASK1 double KO mice) [23,44,45]. These studies demonstrated that deletion of ASK1 decreased oxidative stress levels and increased RGC survival, suggesting that targeting oxidative stress is an effective approach for treatment of glaucoma. Interestingly, it is possible that ASK1 deletion may also have indirect effects on RGC survival, such as by reducing TNF-α production by macrophages, microglia and astrocytes [46,47], in which TNF-α is reported to mediate neurodegeneration in glaucoma [48]. Recently, ASK1 has attracted much attention because of its pathogenic role in non-alcoholic steatohepatitis (NASH), which led to the ASK1 inhibitor selonsertib entering human clinical trials [49,50]. It is intriguing to test the effects of the ASK1 inhibitor on various animal models of glaucoma and explore its therapeutic potential for glaucoma.

### 4.2. Valproic Acid

Valproic acid (VPA) is a short chain fatty acid and it has been used clinically worldwide for treatment of epilepsy since 1970s. Mechanisms of action of VPA are complex and there are multiple pharmacological actions, including increasing GABA synthesis, inhibiting histone deacetylases and neuroprotection [51,52,53]. We reported that VPA suppresses glaucoma-like retinal degeneration in GLAST KO mice by reduction of the oxidative stress level in the RGCs and by stimulation of the BDNF-TrkB pathway [24,54]. Antioxidant properties of VPA have been demonstrated by other groups, for example, in the brain following ischemia/reperfusion injury [55] and in motor neurons following spinal cord injury [56]. It is possible that VPA acts as a histone deacetylase inhibitor and upregulates gene expressions of antioxidant enzymes such as superoxide dismutase and catalase [57]. Intriguingly, some studies reported that oral administration of VPA in patients with retinitis pigmentosa, an inherited retinal dystrophy that is characterized by selective degeneration of photoreceptors, improved visual function, demonstrating clinical efficacy in retinal diseases [58,59,60]. VPA is a drug that is already approved for clinical use in treatment of various conditions with relatively minor side effects. Use of VPA for retinal diseases in clinical settings has not been considered yet, but recent data indicating its therapeutic efficacy in glaucoma and retinitis pigmentosa suggest that VPA is a suitable candidate for ‘drug repositioning’, which is an application of known drugs to new medical conditions to save time and cost that is required to establish the safety of the drug. Findings from numerous studies indicate that VPA may be effective in treatment of glaucoma and retinitis pigmentosa, and further studies are required to determine if it is suitable for treatment of retinal diseases.

### 4.3. N-acetylcysteine

*N*-acetylcysteine (NAC) is a *N*-acetyl derivative of cysteine that has historically been used as an antidote against paracetamol overdose, and more recently for various medical conditions including bronchopulmonary disorders, renal disorders, and neurological and psychiatric disorders. In neurons, the availability of cysteine is the rate-limiting substrate for the synthesis of GSH, a powerful antioxidant, so supply of NAC that can be rapidly hydrolyzed and converted to cysteine can increase GSH levels that may lead to neuroprotection. We have recently reported that daily NAC administration protected RGCs in EAAC1 KO mice by increasing retinal GSH levels and reducing oxidative stress, demonstrating that supplementation of cysteine in neurons via NAC in EAAC1 KO mice restores the retinal GSH levels [26]. These findings demonstrate that NAC exerts neuroprotective effects by its antioxidant properties in EAAC1 KO mice and that NAC may be a potential candidate for glaucoma therapy.

### 4.4. Spermidine

Spermidine is a naturally occurring polyamine and it is vital for life. It has been reported that decrease in the spermidine concentration is associated with aging in humans, and exogenous application of spermidine increased the lifespan of yeast, flies, worms, and human immune cells [61]. Spermidine has been shown to reduces oxidative stress both in vitro and in vivo: spermidine-treated yeast cells and mouse fibroblast cells are less susceptible to damage induced by H_2_O_2_ treatment than non-treated cells, and oral intake of spermidine increases the serum level of free thiol groups in mice [61,62]. We reported that oral intake of spermidine suppresses RGC death and visual impairment in EAAC1 KO mice as well as in the optic nerve injury model, by reducing oxidative stress levels in the retina [28,63]. We found that spermidine suppresses activation of the ASK1-p38 pathway in RGCs and reduces expression of inducible nitric oxide synthase (iNOS) in microglia in an ONI model [63]. These findings demonstrated that oral intake of spermidine exerts antioxidative effects and it is beneficial for glaucoma therapy. Spermidine is a natural component of our diet and studies reported that blood spermidine levels could be increased by eating food that is rich in spermidine, for example, soybeans and mushrooms [64]. Therefore, the beneficial effects of spermidine are easily attainable by choosing the right food.

### 4.5. Coenzyme Q10

Coenzyme Q10 (CoQ10) is an important antioxidant that is mainly located in the inner mitochondrial membrane. The neuroprotective effects of CoQ10 have been reported in neurodegenerative diseases and the retinal level of CoQ10 decreases by 40% with age in humans [65,66]. Studies indicate that when CoQ10 was fed to DBA/2J mice, a mouse model of age-dependent, inherited glaucoma, the RGC survival rate was increased [67]. This protective effect was associated with reduced upregulation of *N*-methyl-d-aspartate receptor subunits NR1 and NR2A, suggesting that CoQ10 may be able to reduce glutamate excitotoxicity as well as oxidative stress in glaucoma eyes. Topical application of CoQ10 also protected RGCs in a rat model of ocular hypertension and mouse model of kainite-induced retinal damage [68,69]. Findings from these animal studies suggested that CoQ10 was worth further investigation for its potential use in treatment of glaucoma patients. Indeed, a clinical study reported that treatment with CoQ10 and vitamin E, in addition to β-blockers, in POAG patients improved pattern electroretinogram and visual evoked potential, indicative of the beneficial effects of CoQ10 and vitamin E on the inner retinal function and visual cortical responses [70]. Further randomized clinical trials in POAG patients to evaluate the effects of CoQ10 and vitamin E in an oral formulation or an eye drop are currently in progress (NCT04038034; NCT03611530) [71]. These trials are looking at the additional effects of CoQ10 and vitamin E to hypotensive drugs and the outcome is awaited.

## 5. Effects of Dietary Intake of Antioxidants in Glaucoma Patients

Several clinical studies suggest that dietary antioxidants may be effective for slowing down progression of glaucoma [72]. Indeed, the association of reduced plasma levels of vitamin C and E with POAG has been indicated [73], and the plasma levels of vitamin E were significantly lower in NTG patients [74]. Studies of African American women aged between 65 and 94 demonstrated that oral consumption of fruits and vegetables that contain high levels of vitamins A and C and carotenoids may be associated with reduced risk of glaucoma [75]. Furthermore, a case study reported that an NTG patient who received dietary supplement containing a mixture of citicoline, homotaurine and vitamin E once a day with a topical brimonidine and brinzolamide drops showed a significant improvement in visual field and stable retinal fiber layer and ganglion cells, suggesting a synergic neuroprotective effect from the dietary supplement [76]. On the other hand, a prospective study indicated that a higher dietary intake of vitamins C, E or A had no effect on risks of glaucoma [77,78]. These contradictory reports suggest that the findings regarding the use of these vitamins for treatment of glaucoma should be taken with caution.

Niacin, also known as vitamin B3, is showing promising results as a therapeutic candidate for glaucoma. Studies from DBA/2J mice demonstrated that oral administration of niacin reduced RGC death [79] and the therapeutic role of niacin in glaucoma was supported by studies of NTG patients indicating that there was a reduced level of dietary niacin intake in NTG patients [80]. Recent clinical trials demonstrated that supplementation with niacin improved inner retinal function in glaucoma patients [81], and the long-term effects of niacin supplementation are under investigation at present.

## 6. Conclusions

Pathogenesis of glaucoma involves multiple factors, but currently available therapies that are clinically effective mainly target reduction of IOP. Research on exploring novel therapeutic strategies that target oxidative stress is increasing, and combinatory treatment of IOP reduction and suppression of oxidative stress may prove effective. Future studies are required to validate the effectiveness of neuroprotection in glaucoma patients, but neuroprotective strategies in addition to IOP-lowering therapy may benefit many glaucoma patients, particularly those who do not achieve sufficient therapeutic effects with IOP reduction alone.
